# Supplementary material for: Emergence of novel methicillin resistant Staphylococcus pseudintermedius lineages revealed by whole genome sequencing of isolates from companion animals and humans in Scotland
Source: PLoS One. 2024 Jul 5;19(7):e0305211. doi: 10.1371/journal.pone.0305211 (PMC11226068; doi:10.1371/journal.pone.0305211)
Supplement: S1 Table — (DOC) [file pone.0305211.s001.doc]

|  | Sequence type (ST) |
| --- | --- |
| MRSP | ST555, ST557, ST560, ST561, ST562, ST569, ST647, ST667, ST668, ST726, ST745, ST1344, ST1520, ST1524, ST1568, ST1885. |
| MSSP | ST554, ST558, ST590, ST591, ST673, ST681, ST682, ST683, ST734, ST735, ST736, ST737, ST738, ST739, ST1342, ST1343, ST1345, ST1515, ST1516, ST1517, ST1518, ST1519, ST1525, ST1526, ST1527, ST1528, ST1529, ST1530, ST1531, ST1618, ST1679, ST1680, ST1681 |

Table S1. Novel STs submitted to pubMLST for assignment
