# Supplementary material for: Emergence of novel methicillin resistant Staphylococcus pseudintermedius lineages revealed by whole genome sequencing of isolates from companion animals and humans in Scotland
Source: PLoS One. 2024 Jul 5;19(7):e0305211. doi: 10.1371/journal.pone.0305211 (PMC11226068; doi:10.1371/journal.pone.0305211)
Supplement: S2 Table — (DOC) [file pone.0305211.s002.doc]

Table S2. Full set of resistance phenotypes for MSSP (n = 321).

| Resistance phenotype | Cat | Dog | Human | Otter | Total |
| --- | --- | --- | --- | --- | --- |
| P | 2 | 175 | 12 | 0 | 189 |
| P, W | 2 | 60 | 2 | 0 | 64 |
| Fully susceptible | 0 | 13 | 3 | 0 | 16 |
| P,E,Da | 0 | 11 | 1 | 0 | 12 |
| P,Te | 0 | 6 | 3 | 0 | 9 |
| Te | 0 | 3 | 2 | 0 | 5 |
| P,E | 0 | 3 | 1 | 0 | 4 |
| P,E,Da,W | 0 | 3 | 1 | 0 | 4 |
| W | 0 | 4 | 0 | 0 | 4 |
| P,Cn,W | 0 | 2 | 0 | 0 | 2 |
| P,Da | 0 | 1 | 1 | 0 | 2 |
| P,E,Da,Ch | 0 | 2 | 0 | 0 | 2 |
| P,Ch | 0 | 1 | 0 | 0 | 1 |
| P,Cn,En,E,Da,W | 0 | 1 | 0 | 0 | 1 |
| P,E,Da,Ch,W | 0 | 1 | 0 | 0 | 1 |
| P,E,Da,Te | 0 | 0 | 1 | 0 | 1 |
| P,E,Da,Te,Ch | 0 | 0 | 1 | 0 | 1 |
| P,En,E,Da | 0 | 1 | 0 | 0 | 1 |
| P,En,W | 0 | 1 | 0 | 0 | 1 |
| P,Te,W | 0 | 1 | 0 | 0 | 1 |

Ch, chloramphenicol; Da, clindamycin; En, enrofloxacin; E, erythromycin;Cn, gentamicin; P, penicillin; Te, tetracycline; W, trimethoprim
