# Supplementary material for: Emergence of novel methicillin resistant Staphylococcus pseudintermedius lineages revealed by whole genome sequencing of isolates from companion animals and humans in Scotland
Source: PLoS One. 2024 Jul 5;19(7):e0305211. doi: 10.1371/journal.pone.0305211 (PMC11226068; doi:10.1371/journal.pone.0305211)
Supplement: S3 Table — (DOC) [file pone.0305211.s003.doc]

Table S3. Full set of resistance phenotypes for MRSP (n = 85).

| Resistance phenotype | Cat | Dog | Human | Otter | Total |
| --- | --- | --- | --- | --- | --- |
| P,Ox,Cn,En,E,Da,Te,W | 4 | 15 | 2 | 0 | 21 |
| P,Ox,Cn,En,E,Da,W | 0 | 9 | 0 | 1 | 10 |
| P,Ox,Cn,E,Da,Te,W | 0 | 8 | 0 | 0 | 8 |
| P,Ox,Cn,En,E,Da,Ch,W | 0 | 8 | 0 | 0 | 8 |
| P,Ox,Cn,En,E,Da,Te,Ch,W | 0 | 6 | 0 | 0 | 6 |
| P,Ox,Cn,E,Da,Ch | 0 | 4 | 0 | 0 | 4 |
| P,Ox,Cn,En,E,Da,Ch | 0 | 3 | 0 | 0 | 3 |
| P,Ox,En,E,Da,W | 0 | 3 | 0 | 0 | 3 |
| P,Ox,Cn | 0 | 2 | 0 | 0 | 2 |
| P,Ox,Cn,E,Da | 0 | 2 | 0 | 0 | 2 |
| P,Ox,Cn,E,Da,Ch,W | 0 | 2 | 0 | 0 | 2 |
| P,Ox,Cn,E,Da,Te,Ch | 0 | 2 | 0 | 0 | 2 |
| P,Ox,Cn,E,Da,W | 0 | 2 | 0 | 0 | 2 |
| P,Ox,Cn,E,Te,Ch | 0 | 2 | 0 | 0 | 2 |
| P,Ox,Cn,E,Te,W | 0 | 2 | 0 | 0 | 2 |
| P,Ox | 0 | 1 | 0 | 0 | 1 |
| P,Ox,Cn,E,Da,Te,Ch,W | 0 | 1 | 0 | 0 | 1 |
| P,Ox,Cn,En,Da,Te,W | 0 | 1 | 0 | 0 | 1 |
| P,Ox,Cn,En,E,Da | 0 | 1 | 0 | 0 | 1 |
| P,Ox,Cn,En,E,Da,Mup,Te,W | 0 | 1 | 0 | 0 | 1 |
| P,Ox,Cn,En,E,Te,W | 0 | 1 | 0 | 0 | 1 |
| P,Ox,Cn,Te,W | 0 | 1 | 0 | 0 | 1 |
| P,Ox,E,Da,Te,W | 0 | 0 | 1 | 0 | 1 |

Ch, chloramphenicol; Da, clindamycin; En, enrofloxacin; E, erythromycin; Cn, gentamycin; Mup, mupirocin; Ox, oxacillin; P, penicillin; Te, tetracycline; W, trimethoprim
