# Supplementary material for: Emergence of novel methicillin resistant Staphylococcus pseudintermedius lineages revealed by whole genome sequencing of isolates from companion animals and humans in Scotland
Source: PLoS One. 2024 Jul 5;19(7):e0305211. doi: 10.1371/journal.pone.0305211 (PMC11226068; doi:10.1371/journal.pone.0305211)
Supplement: Attachment — Submitted filename: Reviewer response.doc [file pone.0305211.s004.doc]

**PONE-D-23-29835 Emergence of novel methicillin resistant *Staphylococcus pseudintermedius* lineages revealed by whole genome sequencing of isolates from companion animals and humans in Scotland**

**Response:** We thank the reviewer for their thoughtful comments and suggestions, which have allowed us to improve this manuscript. Below are our responses to the specific points raised. We have incorporated the resulting changes into the manuscript.

For clarity, in this document, reviewer comments are in italics, with original line numbers. Corresponding line numbers in the revised manuscript are specified as [revised line xx]. Our response to each comment is indented, and modified or new text that is now in the manuscript is underlined.

**Review Comments to the Author:**

Reviewer #1: 
A major concern regards the criteria used for the antimicrobial susceptibility profiling of the isolates (Lines 155-173). It is stated in line 110 (materials and methods) that antimicrobial susceptibility was performed according to EUCAST recommendations. However, EUCAST does not establish zone diameter breakpoints for S. pseudintermedius and several of the antimicrobials tested in this study, such as enrofloxacin (not included in EUCAST recommendations), teicoplanin and vancomycin (glycopeptides only have MIC breakpoints defined). Also, penicillin zone diameter breakpoints are only established for *S. aureus* and *S. lugdunensis*. In addition, for mupirocin, only ECOFF values are given for *S. aureus* when testing a 200 ug mupirocin disc (EUCAST, 2022, v12.0, page 104). Therefore, clarification is needed for readers to understand the categorization of isolates as S or R. Also, in light of these observations, it is suggested a revision of the statement in lines 314-315.

**Response:** *Line 110.* Antimicrobial resistance profile determination changed from	EUCAST to CLSI (VET01S, 2024) guidelines. Fusidic acid and teicoplanin	susceptibilities removed from study. Interpretation of vancomycin susceptibility	was based of Rezaeifar critera (International Journal of Medical Research & Health	Sciences. 2016; 5(11):122–6 [revised lines 121-127]. *Lines 155-173* revised using	CLSI criteria [revised	lines 193-211]. Mupirocin and oxacillin resistance was	determined by the detection of the mecA and mupA genes by PCR [revised lines	197-199]. Lines 314-315 Unchanged [now lines 386-388]

The manuscript describes in detail the population structure of the Scottish S. pseudintermedius, yet its contextualization with AMR phenotypes and genotypes is limited. For instance, similarly to what is occurring in other European countries, a change in the population structure was observed but no discussion on its potential (or absent) impact on AMR is made. For example, are the new lineages associated with different antibiotic resistance phenotypes/genotypes, as already proposed in other studies?

**Response:** Thank you for pointing out this omission. As we have shown a decrease in the prevalence of ST71 MRSP and the emergence of other novel lineages, we compared the rates of resistance, between ST71 and non-ST71 strains, over the study period. This analysis has shown a significantly lower rate of fluoroquinolone resistance in non-ST71 MRSP. No other significant differences were seen between the two groups. Our results and discussion have been amended to reflect this point [revised lines 232-235, 362-370].

It is suggested that the title of the manuscript be shortened, such as "Emergence of novel methicillin resistant Staphylococcus pseudintermedius lineages revealed by whole genome sequencing of Staphylococcus pseudintermedius from companion animals and humans in Scotland".

**Response:** The title of our paper has been shortened, as suggested, to	“Emergence of novel methicillin resistant *Staphylococcus pseudintermedius*

lineages revealed by whole genome sequencing of isolates from companion

animals and	humans in Scotland” [revised lines 1-3].

Other minor concerns/suggestions are detailed below:
*Line 32*: The study by Ruscher et al. (ref #1) focus on the identification and characterization of coagulase positive staphylococci, including *S. pseudintermedius*, from only infection sites in dogs, cats and Equidae; thus, other study/review could be cited to support the statement of the status of *S. pseudintermedius* as a colonizer of dogs, such as ref #3.

**Response:** We have changed the reference as suggested [revised line 40].

*Lines 36 and 39*: Please consider citing the review by Blondeau et al. Zoonotic Staphylococcus pseudintermedius: an underestimated human pathogen? Future Microbiol. 2023 Apr;18:311-315. doi: 10.2217/fmb-2023-0069.

**Response:** Thank you for this helpful suggestion. Reference changed as	suggested [revised lines 46-47].

*Lines 40 – 42/210-217*: This statement is true for MRSP, but not for MSSP for which a wide genetic diversity has been reported in several studies (review ref #3; further corroborated in more recent studies such as the ones by Haenni et al. J Glob Antimicrob Resist. 2020;21:57-59. doi: 10.1016/j.jgar.2020.02.016. and Morais et al. Front Microbiol. 2023;14:1167834. doi: 10.3389/fmicb.2023.1167834.). In addition, I believe ref #8 was exchanged with ref #7.

**Response:** Statement and references amended [revised lines 48-51, 272-281].

*Lines 42 -45/210-217*: It is suggested an update on this statement. Although the decline of predominance of ST71 lineage was first reported in Northern European countries (e.g., ref# 22; ref# 17; ref# 20); in the last three years, several other studies from other European countries have reported a similar trend, including Italy (Nocera et al. Pol J Vet Sci. 2020;23:465-468. doi: 10.24425/pjvs.2020.134693), France (ref# 19) and Portugal (Morais et al. Front Microbiol. 2023;14:1167834. doi: 10.3389/fmicb.2023.1167834).

**Response:** Statement and references amended [revised lines 51-54, 279-281]

*Line 64*: It is not clear from the text and Table 1, which target(s) were used for species identification. Please disclose the target(s) used in the main text.

**Response:** Statement clarified [revised lines 73-74] primers/probe sequences	added to table 1 [revised lines 85].

*Line 72*: The primers for *mupA* detection appear to be missing from Table 1.

**Response:** Primers/probe sequences added to table 1 [revised lines 85].

*Line 89*: Please detail the type of assembly. Did the authors followed a de novo strategy or performed the assembly against a reference strain?

**Response:** A de novo strategy was used. Statement amended [revised line 100].

*Lines 109-114*: EUCAST recommendations do not have breakpoints.

**Response:** We have addressed this major concern by analysing our data against	alternative guidelines [revised lines121-127].

*Lines 116-117*: Please disclose the software used for statistical analysis.

**Response:** Details of the data manipulation, visualisation and statistical analysis	software is now included [revised lines 131-138].

*Line 121*: Please disclose the source of the 85 MRSP isolates.

**Response:** Source of the 85 MRSP included [revised lines145-146]

*Line 122*: Was there any criteria for the selection of the MSSP isolates for WGS?

**Response:** A representative selection of MSSP including all available human isolates were whole genome sequenced	[revised lines 146-147].

*Line 128*: Are there any STs shared by both MRSP and MSSP populations?

**Response:** None of the STs were shared by both populations [revised lines148-	149].

*Lines 131-133*: What was the trend of ST71 detection throughout the time?

**Response:** There was a decreasing trend in the detection of ST71 over the study	period [revised lines 157-159].

*Line 132*: “… and has been [since] detected in seven dogs.”

**Response:** Statement amended [revised line 161-162].

*Line 132*: Please correct to “The second lineage, …”

**Response:** The statement has been amended [revised lines 162].

*Lines 137- 138*: This statement needs clarification. As authors state, analysis of Figure 1 shows that the Scottish *S. pseudintermedius* isolates can be divided in four main clades. However, each clade is not represented by each ST mentioned. For example, ST71, ST68 and ST551 appear to be part of the same clade, independently of the homogeneity degree of the strains that constitute each ST.

**Response:** On the advice of Matt Holden, Professor of genomics at the University	of St Andrews, the statement has been clarified [revised lines 172-176].

*Line 146*: legend of Figure 1: As mentioned previously, the otter isolate was not subjected to the WGS. Therefore, there are no isolates labelled as yellow.

**Response:** Figure 1 is now Figure 2. The Otter isolate (strain name 18-06) is now	included in cgSNP tree [revised line 187].

Tables 3 and 4: It is suggested a different system for organization of antimicrobial resistance phenotypes, either by total number of isolates or by increasing resistance to antimicrobials. From previous results it is deduced that the otter isolate corresponds to a MSSP. However, this isolate (or animals species) does not appear in table 3 or in table 4.

**Response:** Thank you for this suggestion. We have now included these tables as	supplementary material and produced a more detailed representation of the	dominant resistance phenotypes shown in Figure 3. [revised lines 218-226]

*Line 172*: The frequency of MDR isolates should be given also within MRSP and MSSP isolates.

**Response**: We have expanded our results to include a breakdown of the	prevalence of MDR within MRSP and MSSP [revised lines 207-211].

*Lines 198 – 206*: the description of results regarding the presence of antimicrobial resistance genes is poor and should be more detailed. For example, there is no analysis on the distribution of AMR genes and clonal lineages or animal species.

**Response:** We have now included a more detailed description of the presence of	AMR genes in the text [revised lines 245-256] and in figure 5 [revised line 258],	where we show the	relative frequency of each resistance gene, their distribution	over the study period and the prevalence of each resistance genotype by species.

Figure 2: The names qacC, qacD (more rarely) and smr are given in literature to the same gene ([https://tcdb.org/search/result.php?tc=2.A.7.1.1](https://eur01.safelinks.protection.outlook.com/?url=https%3A%2F%2Ftcdb.org%2Fsearch%2Fresult.php%3Ftc%3D2.A.7.1.1&data=05|02|Andrew.Robb@ggc.scot.nhs.uk|87a7dede6777453937a208dc02009792|10efe0bda0304bca809cb5e6745e499a|0|0|638387448909319605|Unknown|TWFpbGZsb3d8eyJWIjoiMC4wLjAwMDAiLCJQIjoiV2luMzIiLCJBTiI6Ik1haWwiLCJXVCI6Mn0%3D|3000|||&sdata=fBW6L4rGOvXgBjHu9gKp63tdEx6b89yU994BEU0aWP4%3D&reserved=0)), which encodes an efflux pump (Smr/QacC/QacD) from the SMR family of transporters, associated with reduced susceptibility to biocides. This gene is located in plasmids and its frequency in S. pseudintermedius is not as high (e.g., Hritcu et al. Front Vet Sci. 2020;7:414. doi: 10.3389/fvets.2020.00414) as the reported in this study. It is suggested that authors verify the frequency of these genes in the genomes analyzed, since it could be related to gene annotations “errors”. For example, a gene could have been annotated as qacC/qacD because it belongs to the SMR family (qacC-like) and not for its high identity with the smr/qacC/qacD gene; in this case, the result should be interpreted with caution.

**Response:** Given this concern, we have removed all reference to these biocide	resistance genes from our study.
*Line 229*: “we detected two lineages, ST551 and ST726, …”

**Response:** This statement has been corrected [revised line 291-92].
*Line 237*: “… no other reports of this lineage…”

**Response:** This statement has been corrected [revised line 297-298].
*Line 240/241*: “… may be a reflection of the shift…”

**Response:** This statement has been corrected [revised line 301].
*Line 230*: ST551 strains have additionally been reported in France (ref #19), Italy (Vitali et al. J Glob Antimicrob Resist. 2021;25:107-109. doi: 10.1016/j.jgar.2021.02.025) and Portugal (Morais et al. Front Microbiol. 2023;14:1167834. doi: 10.3389/fmicb.2023.1167834).

**Response**: This statement has been revised with the appropriate references cited	[revised lines 292-296].
*Lines 253-255/280-292*: Relevant studies have also been published in the last two years. It is suggested an update on the statements and references.

**Response**: These statements and references have now been amended [revised	lines 311-323, 343-352].
*Lines 293-295*: The graphical presentation of some of trends of frequency of AMR (including MRSP/MDR) throughout the 14 years of study would be of interest.

**Response:** A graphical representation of the temporal trend in AMR is shown in	Figure 4 [revised line 237].

- In the references list, there are some italics missing (for example, in lines 346 and 376).

**Response:** Reference have been reviewed and all relevant corrections made.

The authors would, again, like to thank the reviewer for their helpful comments and suggestions.
